# The risk and prognosis of cancer after hospitalisation for herpes zoster: a population-based follow-up study

**DOI:** 10.1038/sj.bjc.6602120

**Published:** 2004-08-24

**Authors:** H T Sørensen, J H Olsen, P Jepsen, S P Johnsen, H C Schønheyder, L Mellemkjær

**Affiliations:** 1 Department of Clinical Epidemiology, Aarhus University Hospital, Vennelyst Boulevard 6, Building 260, 8000 Aarhus C, Denmark; 2 Institute of Cancer Epidemiology, The Danish Cancer Society, Strandboulevarden 49, 2100 Copenhagen Ø, Denmark; 3 Department of Clinical Microbiology, Aalborg Hospital, 9100 Aalborg, Denmark

**Keywords:** herpes zoster, risk, incidence, survival, prognosis, epidemiology

## Abstract

We examined the risk of cancer and survival in a cohort of patients hospitalised with herpes zoster between 1977 and 1996, drawn from the Danish National Registry of Patients. Through linkage with the Danish Cancer Registry, we compared the observed number of cancers with the expected number on the basis of national age-, gender-, and site-specific incidence rates. The survival of herpes zoster patients with cancer was compared with that of non-herpes zoster patients with cancer. Among the 10 588 patients hospitalised with herpes zoster whom we identified, 1427 cancers were observed compared with 1239 expected (relative risk=1.2, 95% confidence interval 1.1–1.2). The risk was substantially elevated during the first year of follow-up, mainly for haematological cancer. Patients with cancer within 1 year of follow-up had a higher prevalence of distant metastases than controls, although the mortality was similar. For those with haematological cancer, however, the mortality was higher for herpes zoster patients than for controls. Haematological cancer following hospitalisation for herpes zoster has a poorer prognosis than in non-herpes zoster patients.

Herpes zoster occurs frequently in immunocompromised patients, such as the elderly and those with lymphoproliferative malignancies, AIDS, and in transplant recipients (Dolin *et al*, 1978; Mazur and Dolin, 1978; Freidman-Kien *et al*, 1986; Melbye *et al*, 1987; Donahue *et al*, 1995; Poulsen *et al*, 1996; Gnann and Richard, 2002). An association between cancer and herpes zoster has been recognised since 1955 (Wyburn-Mason, 1955), and it has been hypothesised that herpes zoster may be a predictor of a subsequent diagnosis of cancer (Smith and Fenske, 1995). Case reports have suggested such an association (Wright and Winer, 1961; Muller, 1967; Cohen *et al*, 1984), but only three relatively small studies have prospectively examined the risk of malignancy subsequent to herpes zoster, and these found no significantly increased risk (Ragozzino *et al*, 1982; Fueyo and Lookingbill, 1984; Wurzel *et al*, 1986). Other studies have examined this association, but most of them were based on patients with prevalent cancer, did not include controls, or were unable to disentangle the temporal relationship (Wyburn-Mason, 1955; McGregor, 1957; de Moragas and Kierland, 1957; Wright and Winer, 1961; Merselis *et al*, 1963; Muller, 1967; Rogers and Tindall, 1971; Schimpff *et al*, 1972; Brown, 1976). In addition, little is known about the prognosis of patients with cancer discovered after hospitalisation with herpes zoster.

To assess the herpes zoster–cancer association without such limitations, we determined the risk of cancer after hospitalisation for herpes zoster, as well as the prognosis of such cancers, using nationwide population-based data from the Danish Registry of Patients and the Danish Cancer Registry.

## METHODS

The Danish National Registry of Patients was established in 1977, and 99.4% of all discharges from Danish nonpsychiatric hospital departments are recorded there (Andersen *et al*, 1999). Recorded information includes the civil registration number, the dates of admission and discharge, the surgical procedures performed, and up to 20 discharge diagnoses. The National Health Service provides tax-supported free medical care to all Danish citizens. The civil registration number, which is unique to every Danish citizen, permits accurate linkage of information among registers. Discharge diagnoses were coded according to the Danish version of the International Classification of Diseases, 8th revision (ICD-8), until 31 December 1993, and according to the 10th revision thereafter (Andersen *et al*, 1999).

All hospitalisations for herpes zoster (ICD-8 codes 053.00–053.9 and ICD-10 codes B02.0-B02.9) from 1 January 1977 to 31 December 1996 were extracted from the National Registry of Patients. We identified 13 414 patients hospitalised with herpes zoster and obtained their full hospitalisation history. This led to the exclusion of 402 patients who had received an organ transplantation before the diagnosis of herpes zoster or were diagnosed with human immunodeficiency virus at any time during the study period, and 2026 patients with a cancer diagnosis prior to or within 2 months after the diagnosis of herpes zoster.

The remaining 10 986 herpes zoster patients were linked to the Central Population Registry for verification of the civil registration number and for information on vital status and emigration. This led to the exclusion of 398 patients who died during the hospitalisation for herpes zoster or had an erroneous registration. The resulting 10 588 eligible herpes zoster patients were classified in two ways on the basis of ICD codes: (1) into 7434 patients with uncomplicated herpes zoster and 3154 patients with complicated herpes zoster; and (2) into 392 patients with disseminated herpes zoster and 10 196 patients with localised herpes zoster.

The 10 588 herpes zoster patients were followed for cancer occurrence from 2 months after the date of discharge with a first time diagnosis of herpes zoster to be certain that the zoster diagnosis came before the cancer diagnosis until (1) the date of death or emigration, identified through the Central Population Registry, (2) a diagnosis of organ transplantation or primary immune deficiency, identified through the National Registry of Patients, or (3) until 31 December 1998, whichever came first.

Information on cancer occurrence was obtained through record linkage to the Danish Cancer Registry with records of all incident cases of cancer in Denmark since 1943. Cancers are classified according to the modified Danish version of the International Classification of Diseases, 7th revision (Storm *et al*, 1997). The registration is based on notification forms that are completed by hospital departments (including departments of pathology and forensic medicine) and practicing physicians whenever a case of cancer is diagnosed or found at autopsy, and whenever changes to the initial diagnosis are made. The cases thus reported manually are supplemented by cases revealed by the computerised linkages to the death certificate file and the National Registry of Patients. Ambiguous or contradictory information, either within a notification form or between forms, leads to requests for clarification in approximately 10% of notifications received. Comprehensive validation has shown that the Registry is 95–98% complete and valid (Andersen *et al*, 1999).

Through the Cancer Registry, we aimed to identify a control group of up to 10 cancer patients without herpes zoster for each herpes zoster patient with cancer. Controls were matched on cancer type, gender, age in 10-year age groups, and calendar year of diagnosis in 5-year periods. However, we were unable to identify 10 matching patients for each herpes zoster patient with cancer, so the control group comprised only 12 193 patients.

### Statistical analysis

The expected number of cancers was calculated on the basis of Danish national incidence rates for primary cancers according to gender, age, and calendar time in 5-year intervals. Multiplying the number of person-years under observation by the appropriate cancer incidence rates yielded the number of cancers that would be expected if patients with herpes zoster had the same risk of cancer as that of the general population. Confidence intervals (CIs) for the standardised incidence ratio – that is, the relative risk, calculated as the ratio of observed to expected cancers – were based on the assumption that the observed number of cases in a specific category follows a Poisson distribution. We examined the association between herpes zoster and all cancer types combined, as well as the association between herpes zoster and haematological cancers, cancers associated with immunosuppression (Nasca, 2001), and other cancers.

Through the Danish Cancer Registry, we obtained information on the extent of cancer spread among the herpes zoster patients who developed cancer during follow-up (cases). The extent of spread was divided into ‘no spread,’ ‘regional spread’, and ‘distant metastases’. We calculated the prevalence ratio of distant metastases (the proportion of cases with distant metastases divided by the proportion of controls with distant metastases) and an associated 95% CI.

We used proportional hazards regression analyses to compare the risk of death after a cancer diagnosis for cases relative to that for controls. We did separate analyses on cases with haematological cancer and those with other types of cancer. The comparisons were based on estimation of mortality ratios and associated 95% CI. All statistical analyses were performed with Stata software (Stata Corporation, Texas, USA).

## RESULTS

The 10 588 herpes zoster patients in the study (3972 men and 6616 women), were followed for 75 774 person-years yielding an average length of follow-up of 7.2 years. The median age at first hospitalisation for herpes zoster was 72 years.

### Cancer risk

We found a total of 1427 cases of cancer with 1239 expected, yielding an overall relative risk of 1.2. (95% CI 1.1–1.2). The relative risks were the same in men and women (data not shown), and did not depend on the year of herpes zoster diagnosis (data not shown).

In the first year of follow-up, 188 cancers were identified, yielding a cumulative cancer risk of 1.8%. Of the 188 cancers, 29 (15%) were haematological cancers, yielding a cumulative risk of haematological cancer of 0.3%. The overall relative risk of a diagnosis of cancer during the first year of follow-up was 1.3 (95% CI 1.1–1.5) and did not vary with the year of hospitalisation. In the first year of follow-up, we found a particularly high risk for haematological cancer with a relative risk of 3.4 (95% CI 2.3–4.9) (Table 1

**Table 1:** Relative risk (RR) for selected cancers within the first year of follow-up among patients previously hospitalised for herpes zoster; follow-up started 2 months after the date of discharge with herpes zoster

	**Observed**	**RR**	**95% CI^a^**
All malignant neoplasms	188	1.3	1.1–1.5
*Haematological cancers*	29	3.4	2.3–4.9
Non-Hodgkin's lymphoma^b^	11	3.8	1.9–6.7
Hodgkin's lymphoma	0	—	—
Multiple myeloma	8	4.8	2.1–9.4
Leukaemia	10	2.8	1.3–5.1
*Immune-related cancers*	32	1.1	0.8–1.6
Liver	3	1.5	0.4–6.0
Cervix	1	0.5	0.01–2.5
Nonmelanoma skin cancer	24	1.1	0.7–1.7
Malignant melanoma	1	0.5	0.01–2.5
Sarcoma^c^	3	2.7	0.6–7.8
*All other sites*^d^	127	1.2	1.0–1.4
Brain	0	—	—
Oesophagus	2	1.3	0.2–5.4
Stomach	6	1.1	0.4–2.3
Colon	12	0.8	0.4–1.4
Rectum	7	1.0	0.4–2.0
Pancreas	6	1.3	0.5–2.7
Lung	22	1.4	0.9–2.2
Breast	12	0.8	0.4–1.4
Corpus uteri	3	0.9	0.2–2.6
Ovary	7	2.3	0.9–4.8
Prostate	14	1.4	0.8–2.4
Kidney	6	1.7	0.6–3.8
Bladder	12	1.4	0.8–2.5

a CI denotes confidence interval.

b Non-Hodgkin's lymphoma is also classified as an immune-related cancer.

c Among sarcomas, only Kaposi's sarcoma is regarded as an immune-related cancer.

d Only selected sites are specified.

).

The relative risk of haematological cancer (Hodgkin's disease, non-Hodgkin's lymphoma, multiple myeloma, and leukaemia) varied with time of follow-up after the herpes zoster diagnosis (Tables 1 and 2

**Table 2:** Relative risk (RR) for selected cancers beyond the first year of follow-up among patients previously hospitalised for herpes zoster; follow-up started 2 months after the date of discharge with herpes zoster

	**Observed**	**RR**	**95% CI^a^**
All malignant neoplasms	1239	1.1	1.1–1.2
*Haematological cancers*	111	1.7	1.4–2.0
Non-Hodgkin's lymphoma^b^	38	1.6	1.1–2.2
Hodgkin's lymphoma	6	3.0	1.1–6.6
Multiple myeloma	22	1.7	1.1–2.6
Leukaemia	45	1.7	1.2–2.2
*Immune-related cancers*	251	1.1	1.0–1.2
Liver	13	1.3	0.7–2.2
Cervix	11	0.8	0.4–1.5
Nonmelanoma skin cancer	203	1.1	1.0–1.3
Malignant melanoma	15	0.8	0.5–1.3
Sarcoma^c^	9	1.1	0.5–2.1
*All other sites*^d^	877	1.1	1.0–1.2
Brain	29	1.6	1.1–2.3
Oesophagus	16	1.5	0.9–2.4
Stomach	44	1.3	0.9–1.7
Colon	97	0.9	0.7–1.1
Rectum	48	0.9	0.7–1.2
Pancreas	39	1.2	0.8–1.6
Lung	130	1.2	1.0–1.4
Breast	127	1.1	0.9–1.3
Corpus uteri	24	1.0	0.6–1.4
Ovary	23	1.1	0.7–1.6
Prostate	79	1.2	0.9–1.4
Kidney	32	1.3	0.9–1.8
Bladder	59	1.0	0.7–1.2

a CI denotes confidence interval.

b Non-Hodgkin's lymphoma is also classified as an immune-related cancer.

c Among sarcomas, only Kaposi's sarcoma is regarded as an immune-related cancer.

d Only selected sites are specified.

), so that it was 4.0 (95% CI 1.3–9.3) in the first 3 months, 3.2 (95% CI 1.4–6.4) between 3 and 6 months, 4.6 (95% CI 2.3–8.2) between 6 and 9 months, 2.1 (95% CI 0.7–4.9) between 9 months and 1 year, 1.9 (95% CI 1.5–2.5) between 1 and 5 years, 1.3 (95% CI 0.9–1.9) between 5 and 10 years, and 1.7 (95% CI 1.1–2.6) after more than 10 years. The relative risks of nonhaematological cancer in the same periods were 1.7 (95% CI 1.2–2.4), 1.5 (95% CI 1.1–1.9), 0.9 (95% CI 0.6–1.3), 0.8 (95% CI 0.5–1.1), 1.1 (95% CI 1.0–1.2), 1.1 (95% CI 1.0–1.2), and 1.1 (95% CI 1.0–1.3), respectively.

For patients with uncomplicated herpes zoster, the relative risk of any cancer during the first year of follow-up was 1.4 (95% CI 1.2–1.6), whereas it was 1.1 (95% CI 0.8–1.5) for patients with complicated herpes zoster. For patients with disseminated herpes zoster, it was 1.5 (95% CI 0.7–2.8), and it was 1.3 (95% CI 1.1–1.5) for patients with other localised herpes zoster.

### Cancer extent and prognosis

Because we were unable to identify matching patients for all 1382 herpes zoster patients with cancer, only 1341 (97%) of these patients were included in the analyses of extent of cancer and of survival after the cancer diagnosis. We had information on the extent of cancer at the time of diagnosis in 79% of cases and 81% of controls. Among those cases who developed cancer within the first year after hospitalisation for herpes zoster, the prevalence ratio of distant spread was higher among the herpes zoster patients than among the controls (prevalence ratio 1.27; 95% CI 0.99–1.63), whereas it was close to unity among those who developed cancer more than 1 year after hospitalisation for herpes zoster (prevalence ratio 1.07; 95% CI 0.92–1.23) (Table 3

**Table 3:** Extent of the spread of cancer according to the presence or absence of herpes zoster

	**Cancer <1 year after herpes zoster**			**Cancer 1–21 years after herpes zoster**		
	**Patients (*N*=176)**	**Controls (*N*=1623)**	**Prevalence ratio (95% CI^a^)**	**Patients (*N*=1165)**	**Controls (*N*=10 570)**	**Prevalence ratio (95% CI^a^)**
Patients with data on spread	136 (77%)	1287 (79%)		923 (79%)	8531 (81%)	
No spread	60 (44%)	607 (47%)		477 (52%)	4692 (55%)	
Regional spread	29 (21%)	330 (26%)		226 (24%)	1914 (22%)	
Distant metastasis	47 (35%)	350 (27%)	1.27 (0.99–1.63)	220 (24%)	1925 (23%)	1.07 (0.92–1.23)

a CI denotes confidence interval.

).

**Figure 1:**
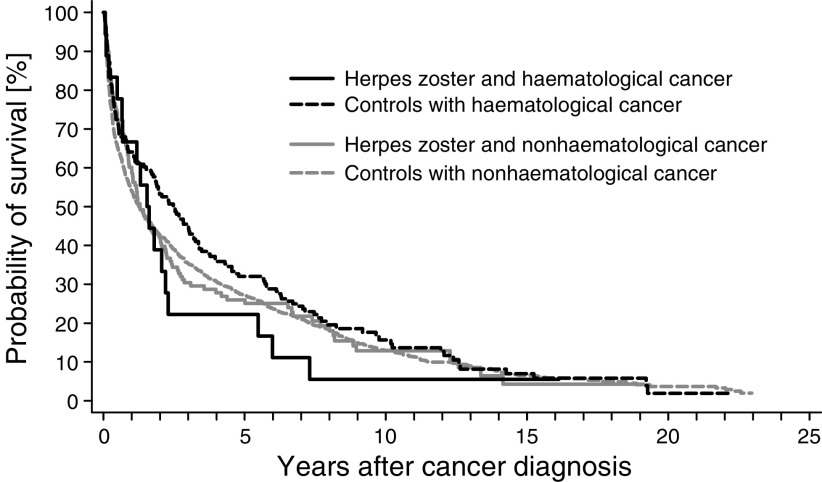
Survival curves for patients with herpes zoster (solid lines) and haematological cancer (black) or nonhaematological cancer (grey). Cancers were diagnosed within the first year of follow-up. Follow-up started 2 months after the date of discharge with herpes zoster. Matched controls are represented by dashed lines.

The survival curves for herpes zoster patients in whom cancer was diagnosed within the first year after herpes zoster hospitalisation, and their matched controls, were almost similar with a mortality ratio of 1.02 (95% CI 0.85–1.22). For cases with haematological cancer, however, the mortality ratio was 1.38 (95% CI 0.83–2.28), whereas that for cases with nonhaematological cancer was 0.98 (95% CI 0.84–1.19) (Figure 1

).

The survival curve for herpes zoster patients in whom cancer was diagnosed more than 1 year after the herpes zoster hospitalisation was similar to that for their matched controls. The mortality ratio was 1.03 (95% CI 0.96–1.11). For cases with haematological cancer, the survival curves for cases and their controls were only slightly different with a mortality ratio of 1.11 (95% CI 0.85–1.46), and for cases with nonhaematological cancer, the mortality ratio was 1.02 (95% CI 0.95–1.11).

## DISCUSSION

In this large, population-based, follow-up study, we found an increased risk of several types of cancer after hospitalisation for herpes zoster, particularly during the first year of follow-up. In particular, there was a strong association between herpes zoster and haematological cancers. Hospitalisation for herpes zoster was also a marker of long-term cancer risk – even after 10 years, we found a 1.7-fold increase in the risk of haematological cancer. Further, patients with herpes zoster were more likely to have advanced cancer than matched controls, although survival curves for nonhaematological cancer were similar. For haematological cancer, however, herpes zoster patients had a poorer survival than their matched controls.

Our findings differ from those of the few previous studies on this topic (Ragozzino *et al*, 1982; Fueyo and Lookingbill, 1984, Wurzel *et al*, 1986). In aggregate, three small studies followed 662 patients. A total of 91 cancer cases were observed compared with 84 expected in the two studies with internal or external controls (Ragozzino *et al*, 1982; Fueyo and Lookingbill, 1984). No cancer cases were found in the third study of children (Wurzel *et al*, 1986). Consequently, these studies had neither the power to detect a substantially increased overall cancer risk nor the ability to study site-specific cancer risks. In addition, they had few clinical details.

There may be several reasons as to why cancer might be associated with herpes zoster. Increased diagnostic effort could explain the association in the short term, but it seems unlikely for heightened surveillance to explain an increased risk many years after the hospitalisation for herpes zoster. Moreover, there was no compensating deficit after a time-limited period of increased risk, and the risk differed between haematological and nonhaematological cancers.

Confounding by smoking may explain a part of the increased risk in the long term since we found an increased risk of many tobacco-related cancers, and smoking, in itself, has some impact on the immune function (Holt, 1987). One common risk factor for both herpes zoster and cancer is cell-mediated immunity, which likely explains the strong associations with haematological cancers. Varicella zoster virus remains in a dormant state until the cell-mediated immune system is depressed and allows the virus to multiply. There is some evidence that infections may promote certain types of cancers (Kuper *et al*, 2000). It has also been suggested that herpes zoster has a direct carcinogenic effect (Rogers and Tindall, 1971).

We found an increased long-term risk of brain tumour after herpes zoster hospitalisation, but it is not possible to assess whether this finding was caused by chance or unknown confounding, or whether it reflects the tropism for the virus towards neural tissue. However, this hypothesis was not supported by the findings of an American case–control study in which adults with glioma were less likely than controls to have had prior varicella zoster virus infection or to have an immunoglobulin-G antibody response adequate to indicate positivity (Wrensch *et al*, 1997; 2001).

The survival curves for herpes zoster patients with nonhaematological cancer and controls were similar indicating that herpes zoster is not associated with particularly aggressive nonhaematological cancer. For haematological cancer, on the other hand, herpes zoster appears to be associated with more aggressive disease, and this is similar to what has been found for other paraneoplastic syndromes, for example, venous thromboembolism and hypercalcaemia (Kristensen *et al*, 1998; Sørensen *et al*, 2000).

The study population was large and well defined, and the long-term follow-up was complete, since our design used computerised registries with complete nationwide coverage of hospitalised patients. Consequently, our findings are limited to a population of severe herpes zoster cases that require hospitalisation, but the risk estimates varied only slightly with the type of herpes zoster patients. Limitations of our study are the existence of coding errors and the possible misclassification of the herpes zoster diagnosis in the National Registry of Patients. If nondifferential, this problem would reduce the strength of the associations we reported. Also, we lacked direct information on potential confounders, such as cigarette smoking and use of immunosuppressive drugs.

Our data clearly showed an association between hospitalisation for herpes zoster and cancer, in contrast to previous research, and even demonstrated a long-term association. Our data are consistent with the hypothesis that herpes zoster may be a marker of haematological cancers, in particular. Nonetheless, the limitations of our data prevent us from suggesting guidelines for searching for occult cancer in patients with herpes zoster. In our cohort, patients diagnosed with cancer in the first 2 months after herpes zoster were excluded, but it is most likely that some cancers diagnosed in the following months were present at the time of hospitalisation for herpes zoster. Early diagnosis of some cancers would have required extensive work-up, and it is unclear whether early diagnosis would have changed the outcome. Some of the haematological cancers might be detected by simple methods, but only 1.8% of herpes zoster patients had a cancer diagnosis within the first year of follow-up, including 0.3% with haematological cancer, and 35% had metastases at the time of diagnosis. These findings suggest that cancer screening of patients hospitalised for herpes zoster will have low efficacy.
